# Chylous aqueous humor caused by hyperlipidemia: A case report and literature review

**DOI:** 10.1097/MD.0000000000034972

**Published:** 2023-09-08

**Authors:** Jing Zhao, He Zou, Jun Xiao

**Affiliations:** a Department of Ophthalmology, The Second Hospital of Jilin University, Jilin University, Changchun, Jilin, P.R. China.

**Keywords:** chylous aqueous humor, CRVO, hyperlipidemia, lipemia retinalis

## Abstract

**Rationale:**

Generally, there is no lipoprotein in aqueous humor, and chyle usually exists transiently in the body. Therefore, persistent chylous aqueous humor is rare.

**Patient concerns:**

We report a case of a 39-year-old man with persistent milky white appearance over the right eye.

**Diagnoses:**

The patient had a history of poorly controlled diabetes for the past 2 years and central retinal vein occlusion of the same eye for the past 2 weeks. The patient’s right eye had a uniform milky appearance in the anterior chamber, transparent cornea, and no keratic precipitate in the posterior cornea. Color Doppler ultrasound of the affected eye showed no obvious inflammation in the vitreous cavity. Laboratory tests revealed severe chylemia. The patient was finally diagnosed as chylous aqueous humor.

**Interventions and outcomes:**

After conventional hypolipidemia and hypoglycemia treatment and locally glucocorticoid treatment. The milky white changes in the anterior chamber improved considerably and finally disappeared.

**Lessons:**

Although the impact of hyperlipidemia on the cardiovascular system and digestive system is well known, its impact on the eyes is often overlooked. We report a rare case of unilateral chylous aqueous humor caused by hyperlipidemia. Through the analysis of this special case, we recommend that ophthalmologists should pay attention to the impact of blood lipid change on eyes.

## 1. Introduction

When mentioning the systemic diseases that negatively impact the eye, we usually think about diabetes and hypertension, but often ignore hyperlipidemia. however, hyperlipidemia is also often overlooked because there are no obvious symptoms or abnormal signs in the early stages of the disease until serious complications arise. In this article, we report a special case of chylous aqueous humor caused by familial hyperlipidemia and review the relevant literature.

## 2. Case presentation

**Figure 1.**
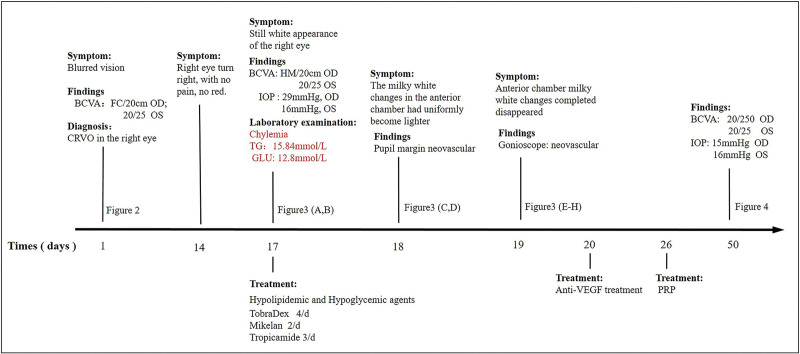
Brief timeline of the patient’s disease course.

A 39-year-old man presented with a milky white appearance over the right eye which had persisted for 3 days. The patient had no visible eye redness and did not report any pain or other symptoms. (The brief timeline is described in Fig. 1.)

**Figure 2.**
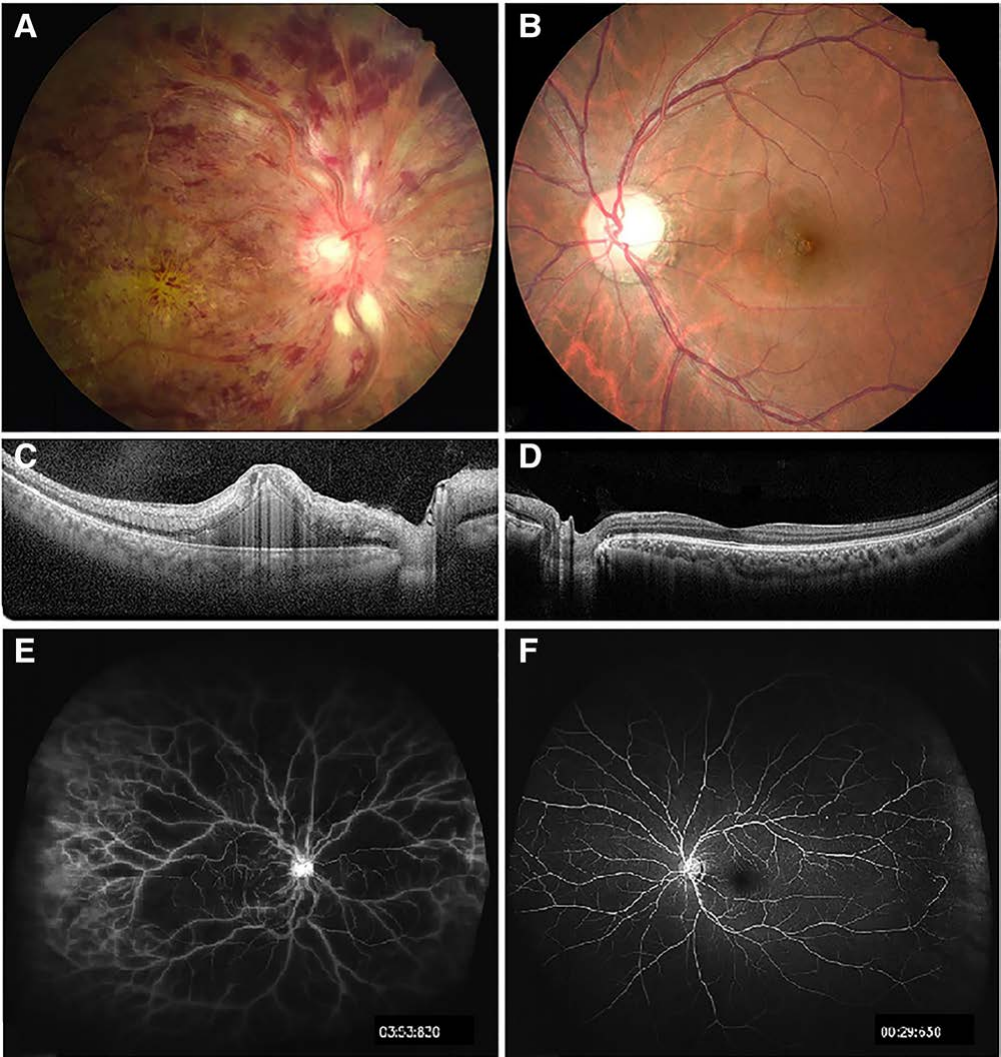
Fundus manifestation of the patient during initial consultation. Fundus photography of the right eye (A) and left eye (B). Images of optical coherence tomography of the right eye (C) and left eye (D). Fluorescein angiography image of the right eye (E) and left eye (F). Diagnostic images show extensive hemorrhage in the fundus, macular edema, and large areas with no perfusion in the right eye. The left eye is normal.

The patient has a 2-year history of poorly controlled diabetes. Two weeks earlier, the patient presented to our hospital with decreased vision in the right eye. Physical examination revealed the following: visual acuity was fingers counting/20 cm for oculus dexter (OD) and 20/25 for oculus sinister (OS); intraocular pressure (IOP) was 14 mm Hg OD and 15 mm Hg OS. The pupil of the right eye was 5 mm, light reflection was slow, and the fundus was bleeding extensively. On evaluation of the left eye, the anterior segment and the fundus was normal. Fundus angiography showed an extensive non-perfusion area and macular edema on the fundus of the right eye (Fig. 2). Based on these findings, the patient was diagnosed with central retinal vein occlusion (CRVO) and macular edema of the right eye. The patient was then scheduled for retinal laser photocoagulation and anti-vascular endothelial growth factor (VEGF) therapy in the right eye in 2 weeks.

**Figure 3.**
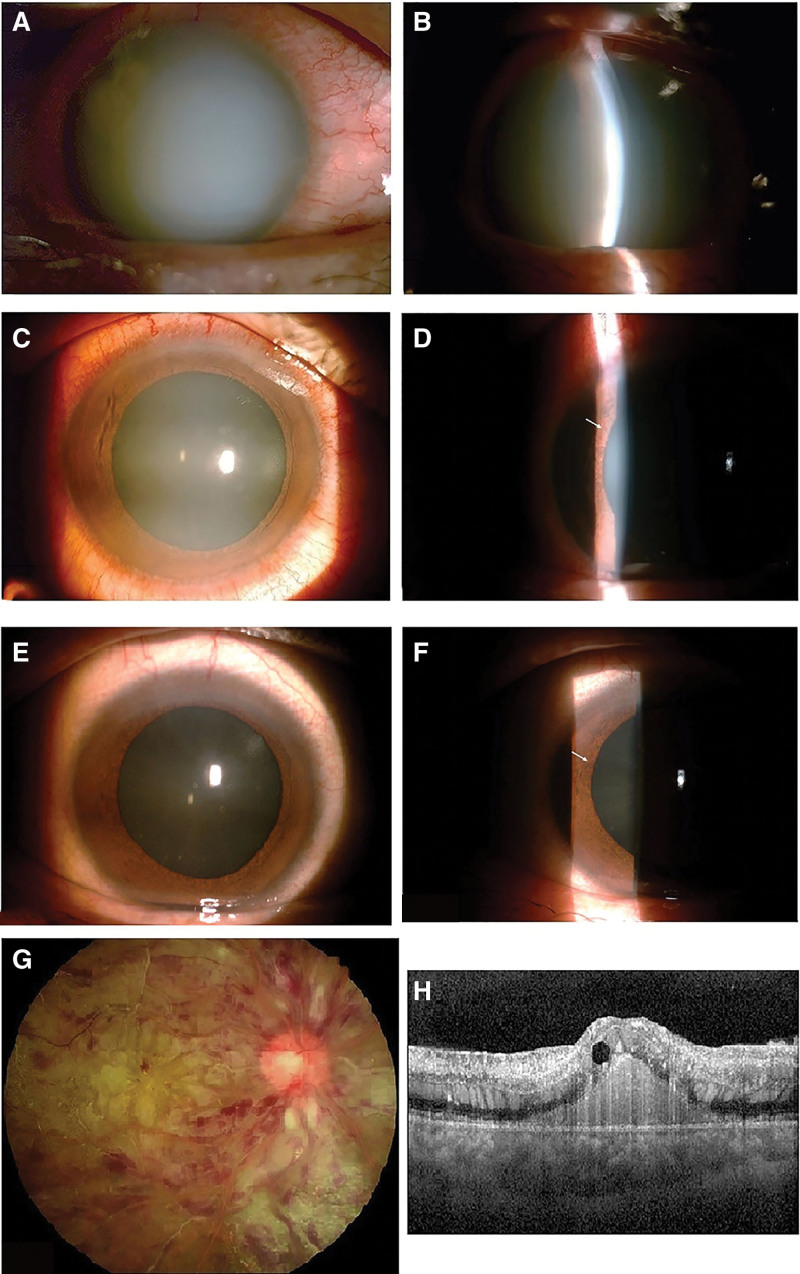
Images of patient’s right eye during the second consultation. (A and B) Slit lamp photographs at the second consultation. Images shows a uniform milky appearance in the anterior chamber which had been present for 3 days, the iris, pupil, and intraocular structure were obscured. (C and D) Slit lamp photographs 1 day post treatment, iris neovascularization was dimly observed at the temporal pupil margin (white arrow). (E–H) Slit lamp, fundus and optical coherence tomography images 2 days post treatment iris neovascularization could be seen clearly at the temporal pupil margin (white arrow).

On the second visit, the patient’s visual acuity was hand movement/20 cm OD and 20/25 OS; IOP was 29 mm Hg OD and 15 mm Hg OS, respectively. Slit lamp examination of the right eye showed a uniform milky appearance in the anterior chamber, smooth and transparent cornea, and no keratic precipitate (KP) in the posterior cornea; the iris, pupil, and intraocular structure were obscured (Fig. 3A and B). The anterior segment of the left eye had no obvious abnormalities. Color Doppler ultrasound of the right eye showed no obvious inflammation in the vitreous cavity. Dilated fundus examination of the left eye showed retinal lipemia. Laboratory examination revealed severe chylemia, triglyceride: 1603.42 mg/dL, fasting blood glucose: 12.8 mmol/L. Other blood test parameters, hepatic function tests, renal function tests, and chest radiography were all unremarkable.

The patient was diagnosed with pseudo uveitis of the right eye, retinal lipemia of the left eye, and hyperlipidemia. We administered acipimox and insulin to control the patient’s blood lipids and blood glucose according to the physician’s advice. Locally we applied tobramycin dexamethasone eye drops and tropicamide eye drops for anti-inflammation, and carteolol eye drops to control the IOP.

The milky white changes in the anterior chamber had uniformly become lighter on the second day (Fig. 3C and D) and had completely disappeared by the third day (Fig. 3E–H). There was still no KP in the posterior cornea or exudation in the anterior chamber. Iris neovascularization was observed at the temporal pupil margin on the right eye on the second day, and anterior chamber angle examination on the third day revealed a large amount of neovascular in the surrounding iris and scleral spur in the right eye, but the chamber angle remained unblocked.

To avoid progression into neovascular glaucoma, we gave anti-VEGF treatment to his right eye the next day after the neovascular in the surrounding iris was observed and performed panretinal laser photocoagulation for his right eye 1 week later.

**Figure 4.**
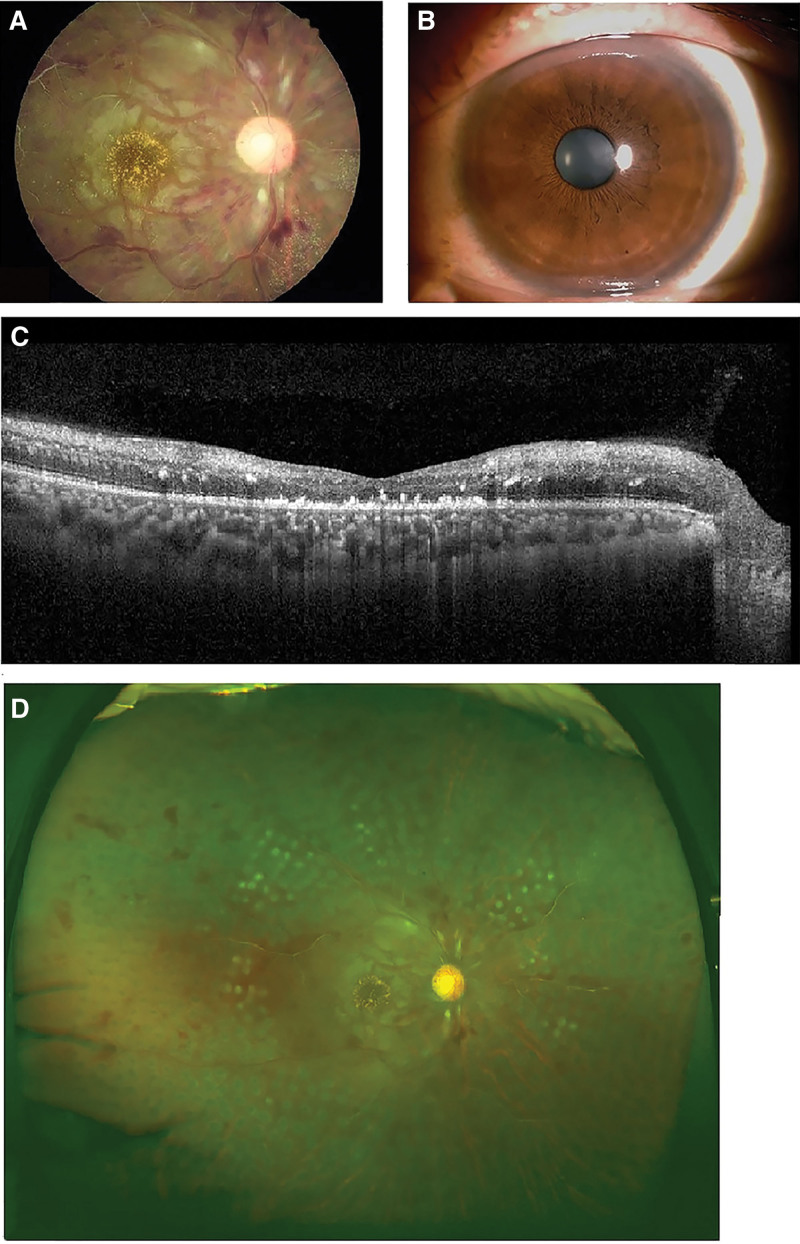
Images of the right eye 1 month after treatment. Fundus photographs (A), slit lamp photographs (B), and optical coherence tomography (C). Images showed normal anterior segment, fundus hemorrhage due to CRVO had absorbed, clear laser spot, pale optic disc, white linear arteries, macular edema subsided and macular retinal structure was atrophic.

Finally, after 3 times of panretinal laser photocoagulation, the patient’s condition was stable, on 1 month follow up, his visual acuity of the right eye was 20/250, the IOP was normal, the milky white change in the anterior chamber did not reappear, the neovascularization of iris and pupil margin had subsided, and the laser spot on the fundus was clear (Fig. 4), the anterior segment and the fundus of the left eye was normal. The patient was followed up for 1 year.

## 3. Discussion

Chylomicrons are the largest of the 5 types of lipoproteins in the human body, with a diameter of approximately 75 to 1000 nm. After the ingestion of fat, triglycerides are bound to lipoproteins by intestinal mucosal cells to form chylomicrons, which then enter the blood stream. When the plasma triglyceride level is > 1000 mg/dL, chylous changes occur in the blood.^[1,2]^ When the plasma triglyceride level > 2000 mg/dL, lipemia retinalis can occur.^[3,4]^ Lipemia retinalis is often overlooked and ignored because it does not affect visual acuity itself.

Usually, there is no lipoprotein in the aqueous humor. The endothelium of iridal vessels and nonpigmented ciliary epithelium prevents the passage of large lipid-laden molecules into the aqueous humor. In our case, the reason for occurrence of chylous aqueous humor was considered related to the damage of the blood-aqueous barrier (BAB). However, unlike the inflammatory anterior chamber changes caused by uveitis, the aqueous humor in this case was uniformly milky, without retrocorneal KP or any fibrin formation in the anterior chamber. And the patient had no complaints other than vision loss. Therefore, we considered that the pathogenesis for this case is not the inflammatory exudation caused by uveitis, but the vascular leakage. The size of chylomicron is obviously smaller than that of cells, proteins or other blood components, and the density of chylomicrons is close to that of aqueous humor, so it can be suspended in aqueous humor after leaking through a small pore size, which makes the anterior chamber present a uniform milk-like appearance.

The reason for this small leakage of the BAB remains unclear. CRVO can cause a release of various inflammatory factors and increase vascular permeability.^[5–7]^ Diabetes can also damage vascular endothelial cells and increase vessel permeability. However, both CRVO and diabetes are common, whereas chylous aqueous humor is rare. Therefore, considering the effect of CRVO or diabetes on blood vessels as the cause of this disease is insufficient.

In addition, chyle usually exists transiently in the body and is common after overeating. Chylous is usually not detected in blood after fasting for 12 hours. Ocular changes caused by chyle should disappear with the disappearance of chyle in the peripheral blood. However, the patient visited our hospital 3 days after noticing that his eyes had turned white, and the symptoms still had not improved. Therefore, besides the special leakage point on the blood vessel mentioned above, abnormal chylous metabolism of the patient is also one of the causes of this disease.

Hyperlipidemia, including primary hyperlipidemia and secondary hyperlipidemia, can be divided into 5 types, according to the different electrophoretic profiles of plasma lipoproteins. Among these, types I and V are characterized by a disruption in chyle metabolism disorder, manifesting as hyperchylomicronemia. Lipemia retinalis is one of its clinical characteristic of types I and V hyperlipidemia.^[8]^ Type V hyperlipidemia is more commonly found in adults and has a mostly polygenic inheritance, with an incidence of approximately 1:600. It can be familial or secondary to various causes, common predisposing factors include poor diet, alcohol ingestion, poorly controlled diabetes, hypothyroidism, and nephrotic syndrome.^[9]^ By inquiring about the patient’s daily life and dietary habits, we found that he liked eating meat, often drank alcohol, suffered from diabetes, and had poor blood glucose control.

Therefore, we consider that hyperchylomicronemia secondary to poorly controlled diabetes, which made the patient chyle metabolism disorder was also one of the reasons for the development of chylous aqueous humor. We speculate that by simply controlling diabetes and using lipid-lowering treatment, the anterior chamber symptoms can also be reduced or eliminated. However, because the affected eye of this patient had a history of CRVO, new blood vessels appeared in the iris and anterior chamber angle. To prevent further progression into secondary glaucoma, we actively applied topical drugs to alleviate the anterior chamber symptoms, treated the patient with anti-VEGF therapy, and performed retinal laser photocoagulation as soon as possible.

After the milk-like pathological changes appeared in the left anterior chamber of the patient, the IOP of the affected eye increased slightly, which was considered to be caused by swelling of trabecular meshwork cells and reduced filtration under the stimulation of chylomicrons and inflammatory factors. The IOP was well controlled after anti-inflammatory and IOP-lowering drugs were given.

Chylous aqueous is rare, to the best of our knowledge, there are only 3 reports available on chylous aqueous humor which are summarized in Table 1.^[10–12]^ All 3 cases previously reported were middle-aged men, with monocular onset, the duration of illness varies from 2 to 5 days, with moderate to severe visual loss, no concomitant symptoms such as eye redness and eye pain. At the time of visit, uniform milk-like changes were observed in the anterior chamber, no KP, no inflammatory exudation and pus were observed in the anterior chamber. The fundus could not be visualized clearly at the time of disease onset. According to the examination of the contralateral eye, 1 case showed retinal lipemia and 1 cases showed mild diabetic retinopathy, 2 case had no obvious abnormalities. Laboratory examination showed that in addition to significantly increased serum triglyceride, all the patients had poorly controlled diabetes. All patients were treated with local glucocorticoid, systemic hypoglycemic and lipid-lowering drugs, the symptoms completely disappeared in about 3 days.

**Table 1:** Available reports on chylous aqueous humor.

No.	Age	Gender	Affected eye	Past medical history	The other eye	Follow-up	References
1	32	Male	OD for 3d	DM, schizophrenic	Lipemia retinalis	None	^[10]^
2	43	Male	OD for 2d	DM, hypertension, gout, pyelourinary	Normal	IOP of right eye increased 2 weeks later	^[11]^
3	44	Male	OS for 5d	DM, hypertension, obesity	Mild DM change	CRVO in left eye, 6 weeks later	^[12]^.
Our case	39	Male	OD for 3d	DM, CRVO in OS	Normal	None	

As for the pathogenesis, we do not have a reasonable explanation for the small leakage of the BAB. Based on the special appearance of the affected eyes, we speculate that the small leakage is a response of the vessel endothelium to high concentration of lipoproteins rather than damage induced by inflammatory processes. Some reports have been made about the effect of lipoproteins on endothelial cells,^[13,14]^ but whether a high concentration of lipoproteins has an effect on iridial vessel endothelial cells and nonpigmented ciliary epithelial cells remains to be determined.

Although the impacts of hyperlipidemia on the cardiovascular system and digestive system are well known, the impact on the eyes are often neglected. Hyperlipidemia can lead to a variety of ocular abnormalities. In addition to the special chylous aqueous humor and lipemia retinalis in this case, hyperlipidemia also increased the risk of glaucoma,^[15]^ related to the severity of diabetic retinopathy, hyperlipidemia is one of the main risk factors for CRVO,^[16]^ and had an impact on the development and progression of age-related macular degeneration.^[17]^ More importantly, patients with hyperlipidemia have increased systemic risks of coronary artery disease, myocardial infarction, and stroke. Therefore, the diagnosis and treatment of hyperlipidemia-related ophthalmic diseases are not only important from an ophthalmological point of view but also for some serious potential genetic and metabolic disorders. Timely diagnosis and management of hyperlipidemia can reverse most symptoms and prevent life-threatening complications.^[1]^

## Acknowledgments

We thank the patient for agreeing to the publication of this report.

## Author contributions

**Conceptualization:** Jun Xiao.

**Data curation:** Jing Zhao, He Zou.

**Project administration:** Jing Zhao, Jun Xiao.

**Supervision:** He Zou, Jun Xiao.

**Writing – original draft:** Jing Zhao.

**Writing – review & editing:** Jun Xiao.
